# Antioxidant and Antiulcerogenic Activity of the Dry Extract of Pods of *Libidibia ferrea* Mart. ex Tul. (Fabaceae)

**DOI:** 10.1155/2019/1983137

**Published:** 2019-11-20

**Authors:** Lady D. K. T. Prazeres, Ticiana P. Aragão, Samara A. Brito, Cynthia L. F. Almeida, Amanda D. Silva, Mirella M. F. de Paula, Juliane S. Farias, Leucio D. Vieira, Bolívar P. G. L. Damasceno, Larissa A. Rolim, Bruno O. Veras, Ismael G. Rocha, Jacinto C. Silva Neto, Milena L. F. Bittencourt, Rita de Cássia R. Gonçalves, Rodrigo R. Kitagawa, Almir G. Wanderley

**Affiliations:** ^1^Department of Pharmaceutical Sciences, Universidade Federal de Pernambuco, Recife 50740-521, Brazil; ^2^Department of Nutrition, Universidade de Pernambuco, Petrolina 56328-903, Brazil; ^3^Santa Maria College, Cajazeiras 58900-000, Brazil; ^4^Collegiate of Nursing, Universidade Federal do Vale do São Francisco, Petrolina 56304-205, Brazil; ^5^Department of Physiology and Pharmacology, Universidade Federal de Pernambuco, Recife 50670-901, Brazil; ^6^Department of Pharmacy, Universidade Estadual da Paraíba, Campina Grande 58429-600, Brazil; ^7^Drugs, Medicines and Food Analysis Center, Universidade Federal do Vale do São Francisco, Petrolina 56304-917, Brazil; ^8^Department of Tropical Medicine, Universidade Federal de Pernambuco, Recife 50670-901, Brazil; ^9^Department of Histology and Embryology, Universidade Federal de Pernambuco, Recife 50670-420, Brazil; ^10^Graduate Program in Pharmaceutical Sciences, Universidade Federal do Espírito Santo, Vitória 29047-105, Brazil

## Abstract

Ethnomedicinal studies in the Amazon community and in the Northeast region of Brazil highlight the use of *Libidibia ferrea* fruits for the treatment of gastric problems. However, there are no data in the literature of this pharmacological activity. Thus, the aim of this paper is to provide a scientific basis for the use of the dry extract of *L. ferrea* pods (DELfp) for the treatment of peptic ulcers. Phytochemical characterization was performed by HPLC/MS. *In vitro* antioxidant activity was assessed using DPPH, ABTS, phosphomolybdenum, and superoxide radical scavenging activity. The gastroprotective activity, the ability to stimulate mucus production, the antisecretory activity, and the influence of -SH and NO compounds on the antiulcerogenic activity of DELfp were evaluated. The healing activity was determined by the acetic acid-induced chronic ulcer model. Anti-*Helicobacter pylori* activity was investigated. HPLC/MS results identified the presence of phenolic compounds, gallic acid and ellagic acid, in DELfp. The extract showed antioxidant activity *in vitro*. In ulcers induced by absolute ethanol and acidified ethanol, the ED_50_ values of DELfp were 113 and 185.7 mg/kg, respectively. DELfp (100, 200, and 400 mg/kg) inhibited indomethacin-induced lesions by 66.7, 69.6, and 65.8%, respectively. DELfp (200 mg/kg) reduced gastric secretion and H^+^ concentration in the gastric contents and showed to be independent of nitric oxide (NO) and dependent on sulfhydryl (-SH) compounds in the protection of the gastric mucosa. In the chronic ulcer model, DELfp reduced the area of the gastric lesion. DELfp also showed anti-*H. pylori* activity. In conclusion, DELfp showed antioxidant, gastroprotective, healing, and antiulcerogenic activities. The mechanism of these actions seems to be mediated by different pathways and involves the reduction of gastric secretion and H^+^ concentration, dependence on sulfhydryl compounds, and anti-*H. pylori* activity. All these actions support the medicinal use of this species in the management of peptic ulcers.

## 1. Introduction

Peptic ulcer (PU) is a term used to refer to an acid-peptic lesion of the gastrointestinal tract resulting in rupture of the mucosa and submucosa [1]. Ulcerative lesions occur due to an imbalance between the cytoprotective factors of the mucosa, such as the mucus-bicarbonate barrier and the presence of prostaglandins, and aggressive factors, among them reactive oxygen species (ROS) and hydrochloric acid [2, 3]. It is estimated that 10 to 20% of PU cases are associated with comorbidities such as hemorrhages, obstructions, and perforations, with a mortality rate ranging from 10 to 40%, with 2 to 14% of these mortality cases due to perforation [4–8]. Given this scenario, the scientific community has been continually challenged to contribute research to this area [2].

Several behavioral factors culminate in the development of PUs. They include inadequate eating habits, alcohol and tobacco consumption, stress, and the inadvertent use of nonsteroidal anti-inflammatory drugs (NSAIDs) [9]. In these situations, the defense mechanisms of the gastric mucosa, such as mucus and bicarbonate secretion, acid-base balance, endogenous sulfhydryl groups, and epidermal growth factor [9, 10], are insufficient to maintain tissue homeostasis, favoring the development of lesions.

Oxidative stress increases the formation of ROS such as superoxide anion (O_2_^−^), hydroxyl radical (OH^−^), and hydrogen peroxide (H_2_O_2_) [11]. Excess of ROS harms cellular proteins [12] and disrupts the gastrointestinal tract barrier, increasing tissue permeability, which contributes to inflammation. ROS also cause inflammation by stimulating polymorphonuclear leukocytes, enhancing tissue damage [13].

PU therapy evolved with the advent of gastric acid secretion inhibitors, antihistamines (H_2_), and proton pump inhibitors [14]. Despite the advances, these pharmacological classes have adverse effects. Antihistamines may cause arrhythmia, gynaecomastia, and hematological alterations [15], and treatment with proton pump inhibitors may provoke hyperplasia of the parietal cells of the gastric glands [16].

In addition to adverse effects, other factors such as the nonresponsiveness to pharmacological treatment presented by some individuals should be considered [17], as well as the gastric harm and increased risk of complications such as bleeding and gastric or duodenal perforation in the elderly associated with the use of acetylsalicylic acid (AAS), a nonselective inhibitor of the cyclooxygenase-2 enzyme, often used in the prophylaxis of cardiovascular diseases [18, 19].

Warren and Marshall [20] first described, in 1983, *Helicobacter pylori* as the etiologic factor of peptic ulcer. Despite the numerous studies, the survival of these bacteria in the acidic pH of the gastric lumen is still uncertain. However, bacterial growth in intimate contact with the epithelium, presumably near the neutral end of this gradient, favors bacterial existence due to protection of the overlying mucus [20, 21]. About 90% of patients with chronic gastric lesions are infected with *H. pylori.* Alam et al. [22] and Khulusi et al. [23] showed an association between the concentration of these bacteria and the severity scores of the lesions.

*Libidibia ferrea* (Mart. ex Tul.) L. P. Queiroz var. *ferrea*, previously classified as *Caesalpinia ferrea* Mart. ex. Tul. var. *ferrea*, belongs to the Fabaceae family. It is popularly known as “pau-ferro” or “jucá” [24, 25] and is well represented in the semiarid regions of North and Northeast Brazil [26].

Ethnopharmacological studies in Amazonian communities in Brazil reported the use of *C. ferrea* in the form of tea and syrup and the infusion of the fruits for control of gastric problems [27].

Several biological activities of *L. ferrea* have been scientifically proven, including anti-inflammatory [28, 29], analgesic [28, 30], antibacterial [27], antihypertensive [31], and antidiabetic [26] activities. Guerra et al. [32] associated the activity of this species against lineages of colorectal cancer cells to the increase in glutathione (GSH) levels and reduction of lipid peroxidation.

Various studies with *L. ferrea* have shown the presence of bioactive compounds with antioxidant activity, important for the treatment of several diseases. Considering the use of this species in ethnomedicine, the lack of information in the literature, and the need to propose a therapeutic tool that may help in the treatment of PUs, this study investigated the antioxidant and antiulcerogenic activities of the pods, the renewable part of the plant, of *L. ferrea*.

## 2. Materials and Methods

### 2.1. Plant Material and Extract Preparation

*Libidibia ferrea* pods were collected in the municipality of Barbalha, Ceará, Brazil (7°18′40^″^S, 39°18′15^″^W). A representative sample of the species was deposited in the Herbarium of the Agronomic Institute (IPA) under accession number 90603. The pods were dried at room temperature for 72 h and then ground in a knife mill (Wyllie Macro-TE 650). The plant material (100 g) was subjected to cold maceration with 40% hydroalcoholic solvent for 3 days. After this procedure, the extract was filtered and the solvent completely removed with the aid of a spray dryer (MSD 0.5, LabMaq, Ribeirão Preto, Brazil). The parameters used were as follows: drying air flow, 4.5 m^3^/min; outlet temperature, 95°C; compressed air flow rate, 40 L/min; and peristaltic pump, 0.5 L/h. The dry extract of *L. ferrea* pods (DELfp) presented a yield of 18.9% (*w*/*w*). For the *in vivo* experiments, the extract was diluted in distilled water at the moment of use.

### 2.2. Phytochemical Study

#### 2.2.1. High-Performance Liquid Chromatography (HPLC-MS)

The dry extract of *L. ferrea* pods was subjected to high-performance liquid chromatography coupled to mass spectrometry (LC-MS) using an octadecylsilane (250 × 4.6 mm, 5 *μ*m, Luna C18, Phenomenex) column. The stationary phase and mobile phase were composed of 2 solvents: A—ultrapure water containing 0.1% formic acid and B—methanol containing 0.1% formic acid (HPLC grade) at a flow rate of 1.0 mL/min under the following gradient conditions: 95% to 5% A (0-60 min), 5% to 0% A (60-70 min), and 0% to 95% A (70-90 min). The stationary phase was maintained at 30°C, and the volume injected was 20 *μ*L for the sample (1 mg/mL) monitored at 190 to 400 nm and 50 to 1000 *m*/*z*. The analysis used LC-20 (Shimadzu, Kyoto, Japan) equipped with the quaternary bomb system model LC-20ADVP, degasser model DGU-20A, PDA detector model SPD-20AVP, oven model CTO-20ASVP, automatic injector model SIL-20ADVP, and SCL-20AVP system controller coupled to an ESI-IT mass spectrometer (Bruker Daltonics, Billerica, MA, USA), equipped with an electrospray ionization source operating in the analyzer mode and by positive ion trap. The following mass spectrometer parameters were used: capillary voltage, 3.5 kV; desolvation temperature, 320°C; gas flow, 10 L/min; and pressure, 60 PSI, using nitrogen as drying and misting gas.

### 2.3. Quantification of Secondary Metabolites in DELfp

#### 2.3.1. Determination of Total Phenol Content

The total phenol content (TPC) of DELfp was determined using the Folin-Ciocalteu (FC) reagent, as described by Li et al. [33]. Gallic acid (GA) at concentrations of 31.25, 62.5, 125.0, 250.0, 500.0, and 1000 *μ*g/mL was used to obtain the standard calibration curve (in triplicate). The results were expressed as mg equivalents of AG/g extract (mg EAG/g).

#### 2.3.2. Determination of Flavonoid Content

The flavonoid content of DELfp was determined according to Woisky and Salatino [34]. A 0.5 mL aliquot of the 0.5 mL extract was added to 0.5 mL of 2% AlCl_3_ (*w*/*v*) solution prepared in methanol. After 30 minutes of incubation at room temperature, protected against light, the absorbance at 420 nm was measured. Quercetin (Q) at the concentrations of 31.25, 62.5, 125.0, 250.0, 500.0, and 1000 *μ*g/mL was used to obtain the standard calibration curve (in triplicate). Total flavonoid content was expressed as mg quercetin equivalents/g extract (mg EQ/g).

### 2.4. *In Vitro* Antioxidant Activity of DELfp

#### 2.4.1. ABTS (2′,2-Azino-bis (3-ethylbenzothiazoline-6-sulfonate)) Radical Scavenging Activity

The ABTS scavenging activity was performed according to Uchôa et al. [35], using Trolox as a standard. All experiments were performed in triplicate. The inhibition percentage (*I*%) was calculated considering Equation (1), where *A*_c_ is the absorbance of the control and *A*_s_ is the absorbance in the presence of the extract. The concentration required to eliminate 50% of ABTS radicals (IC_50_) was calculated.

(1) $$\begin{array}{c} I\%=\left[\frac{A_{\text{c}}-A_{\text{s}}}{A_{\text{c}}}\right]\times 100. \end{array}$$

#### 2.4.2. DPPH (2,2-Diphenyl-1-picrylhydrazyl) Radical Scavenging Activity

Free radical scavenging activity was measured by means of hydrogen donation using the stable radical DPPH, using Trolox as a standard [36]. All experiments were performed in triplicate. The inhibition percentage (*I*%) was calculated considering Equation (2), where *A*_c_ is the absorbance of the control and *A*_s_ is the absorbance in the presence of the extract. The concentration required to eliminate 50% of DPPH free radicals (IC_50_) was calculated.

(2) $$\begin{array}{c} I\%=\left[\frac{A_{\text{c}}-A_{\text{s}}\;}{A_{\text{c}}}\right]\times 100. \end{array}$$

#### 2.4.3. Total Antioxidant Activity (TAA)

Total antioxidant activity (TAA) was performed according to the Prieto methodology [37], based on the reduction of molybdenum (VI) to molybdenum (V), in the presence of antioxidants in acid pH. All experiments were performed in triplicate. The TAA of the samples was expressed in relation to ascorbic acid (AA, 0-1000 *μ*g/mL) used as a standard, whose reference antioxidant activity was considered equal to 100%, using Equation (3), where *A*_s_ is the absorbance of the sample, *A*_c_ is the absorbance of the control (blank: without extract), and *A*_aa_ is the absorbance of ascorbic acid. The concentration required for 50% of activity (IC_50_) was calculated.

(3) $$\begin{array}{c} \%\text{TAA}=\frac{\left(A_{\text{s}}-A_{\text{c}}\right)\times 100}{A_{\text{aa}}-A_{\text{c}}}. \end{array}$$

#### 2.4.4. Superoxide Radical (O_2_^−^) Scavenging Activity

This test was performed according to Dasgupta and De [38], with modifications. Superoxide radicals were generated using the system nitrotetrazolium blue chloride/nicotinamide adenine dinucleotide (NBT/NADH). All experiments were performed in triplicate. The inhibition capacity (%) of the photochemical reduction of NBT was calculated using Equation (4), where *A*_c_ is the absorbance of the control and *A*_s_ is the absorbance of the sample. The concentration required to eliminate 50% of radical superoxide (IC_50_) was calculated.

(4) $$\begin{array}{c} \%I=\left[\frac{A_{\text{c}}-A_{\text{s}}}{A_{\text{c}}}\right]\times 100. \end{array}$$

### 2.5. Animals

Three-month Wistar rats (*Rattus norvegicus* var. *albinus*), with body weight between 200 and 330 g, of both sexes were used in the study. The rats were provided by the Department of Physiology and Pharmacology of the Federal University of Pernambuco, Brazil. The animals were housed in cages with open-mesh floors to avoid coprophagy and maintained under controlled lighting (12 h light/dark cycle), temperature (22 ± 2°C), and humidity (55-65%) conditions, receiving diet (Presence, Purina, São Paulo, Brazil) and water *ad libitum*. In all protocols, the animals underwent gavage and were euthanized in a CO_2_ chamber (by inhalation). The number of animals was distributed inhomogeneously to form the different groups considering that during the experimental procedures, intercurrences can occur that result in the death of the animal or its exclusion from the group. The experimental protocols were submitted to and approved by the Ethics Committee on Animal Experimentation of the Federal University of Pernambuco (registration number 0016/2016) according to the Guide for the Care and Use of Laboratory Animals of the National Institute of Health (Washington, DC, 2011).

### 2.6. Acute Toxicity Study in Rats

The acute toxicity study was performed in female Wistar rats, as described by the Organization for Economic Co-operation and Development (OECD 425) [39], with minor modifications. The animals were randomly divided into two groups (*n* = 3/group) and deprived of feed for 12 h with free access to water. Then, the control group received the vehicle (0.9% NaCl solution, 10 mL/kg), and the treated group received a single oral dose of DELfp (2000 mg/kg). After administration, the animals were observed individually at intervals of 30, 60, 120, 180, 240, 300, and 360 min every day for 14 days. The following parameters were evaluated: behavioral changes, clinical signs of toxicity, water and feed intake, and body weight variation.

### 2.7. Gastroprotective Activity

#### 2.7.1. Ethanol-Induced Ulcer

After 16 h of fasting, the animals received, orally, 0.9% NaCl solution (10 mL/kg, negative control, *n* = 6), pantoprazole (40 mg/kg, *n* = 7), or DELfp (50, 100, 200, and 400 mg/kg, *n* = 6). After 1 h, all groups received the ulcerogenic agent Merck absolute ethanol (≥99.9%, 1 mL, orally), according to the method described by Robert et al. [40], with slight modifications. The animals were euthanized in a CO_2_ chamber 1 h after induction of the lesions. The stomachs were removed and photographed, and the gastric lesion area was determined using the ImageJ software version 1.4 (Bethesda, MD, USA). The results were expressed as the area of ulcerative lesion (mm^2^). The effective dose (ED_50_) that inhibited 50% of the ulcerative lesion area was calculated. The glandular portion of the stomachs was used for the determination of lipid peroxidation and nonprotein sulfhydryl compounds and histological analysis.

#### 2.7.2. Determination of Lipid Peroxidation and Levels of Nonprotein Sulfhydryl Compounds

The lipid peroxidation index was determined by measuring the levels of thiobarbituric acid reactive substances (TBARS) using the method described by Ohkawa et al. [41], with modifications. The tissues were homogenized in a solution of potassium chloride (1.15% KCl) and 3 mM EDTA (5 mL of solution/gram of tissue) and kept in an ice bath. Then, 10 mg of the tissues was added to the reaction medium containing sodium dodecyl sulfate (0.4% SDS), 7.5% acetic acid, and thiobarbituric acid (0.3% TBA) (pH 3.5). The reaction tubes were sealed and incubated at 95°C for 60 minutes. After cooling under running water, 1 volume of butanol was added for each volume of the reaction solution, and the tubes were centrifuged at 4000 rpm for 10 minutes. The absorbance of the organic phase was measured at 535 nm. The results were corrected for the protein concentration of the homogenate and expressed in nmol of malondialdehyde (MDA)/mg protein.

The levels of nonprotein sulfhydryl (-SH) compounds in the gastric mucosal homogenate were determined using the methodology of Sedlak and Lindsay [42]. The tissue sample from the homogenate obtained for the evaluation of lipid peroxidation (final concentration 50 mg/mL) was precipitated in trichloroacetic acid solution (5% TCA). The supernatant (final concentration 5 mg/mL) was then added to the reaction medium containing 400 mM TRIS, 4 mM EDTA, and 400 *μ*M 5,5′-dithio-bis-(2-nitrobenzoic acid) (DTNB) (pH 8.9). The reaction was incubated at room temperature for 5 minutes, and the absorbance was measured at 412 nm. The result was corrected for the protein concentration of the homogenate, and the concentration of -SH compounds was expressed in nmol of GSH/mg protein.

#### 2.7.3. Histological Analysis

For this analysis, the stomachs were sectioned and fixed in 10% buffered formalin. Once fixed, the samples were washed with water, immersed in 70% ethyl alcohol for 3-4 days, and embedded in paraffin. 5 *μ*m thick paraffin sections were stained and fixed with hematoxylin/eosin (H&E). Histological analysis of the gastric sections was performed using a MICRO DIP automated microscopy system.

#### 2.7.4. Acidified Ethanol- (Ethanol/HCl) Induced Ulcer

After 16 hours of fasting, the animals received, orally, 0.9% NaCl solution (10 mL/kg, negative control, *n* = 6), pantoprazole (40 mg/kg, *n* = 7), or DELfp (100, 200, and 400 mg/kg, *n* = 6). After 1 h, all groups received the ulcerogenic agent, 1 mL of 0.3 M HCl solution/60% ethanol, orally, according to the method described by Mizui and Douteuchi [43]. The animals were euthanized 1 h after the induction of the lesions. The stomachs were removed and photographed, and the gastric lesion area was determined using the ImageJ software version 1.4 (Bethesda, MD, USA). The results were expressed as the area of ulcerative lesion (mm^2^). The effective dose (ED_50_) that inhibited 50% of the ulcerative lesion area was calculated.

#### 2.7.5. Indomethacin-Induced Gastric Ulcer

After 16 hours of fasting, the animals were given, orally, 0.9% NaCl solution (10 mL/kg, negative control, *n* = 6), pantoprazole (40 mg/kg, *n* = 6), or DELfp (100, 200, and 400 mg/kg, *n* = 7). After 30 minutes of pretreatment, gastric lesions were induced with indomethacin (30 mg/kg subcutaneously) according to the method described by Djahanguiri [44], with modifications. After 6 h of induction, the stomachs were removed and photographed for the determination of gastric lesions, as previously described.

### 2.8. Evaluation of Protective Factors of the Gastric Mucosa

Unlike previous gastroprotective protocols, in which three doses were used to show a dose-effect relation of DELfp, in the experiments below, it was aimed at determining the possible mechanism of action. In this sense, the influence of the indicators on the effect of the extract was evaluated: mucus concentration, gastric content, pH, total acidity, and NO and -SH compounds. Only one dose of DELfp (200 mg/kg) representing the intermediate dose used in the study was selected, since we suppose that to investigate the mode of action of the extract, it is not necessary to use different doses as the pharmacological mechanism of action tends to be similar to that of different doses of the extract.

#### 2.8.1. Determination of Mucus Concentration in the Gastric Mucosa

Soluble glycosaminoglycan content in the gastric mucosa was investigated using Alcian blue, specific for acid mucins [45]. This experiment was performed according to Raffatullah et al. [46] with slight modifications. After 16 hours of fasting, the animals received, orally, 0.9% NaCl solution (10 mL/kg, negative control, *n* = 7), carbenoxolone (200 mg/kg, *n* = 6), or DELfp (200 mg/kg, *n* = 8). A noninjured control group (*n* = 6) was used in this experiment; animals of this group did not receive treatment but underwent surgical stress. After 1 hour, under anesthesia (xylazine, 6 mg/kg associated with ketamine, 60 mg/kg, i.p.), the animals were subjected to longitudinal incision for pyloric ligation. After 4 hours, the animals were euthanized, and the esophagus was pinched off so that stomach contents were not lost. The stomachs were removed, washed with distilled water, dried, and opened along the greater curvature, and the gastric contents were collected and weighed. The glandular portion of the stomach was then weighed and stored in a single vessel immersed in 10 mL of 0.1% Alcian blue solution. After 2 hours of soaking, the excess dye was removed from the glandular portion of the stomach in two successive washes with 7 mL of 0.25 mol/L sucrose for 15 and 45 minutes. After that, the dye complexed with the gastric wall mucus was removed by sequentially transferring each stomach to 10 mL MgCl_2_ (0.5 mol/L) and vortexing them for 1 minute at 30-minute intervals, for 2 hours. The stomachs were removed, and the Falcon tubes with the solution were stored in the refrigerator for approximately 8 h. After this interval, the tubes were shaken for 1 minute, then collected and added to 4 mL of ethyl ether, and vigorously vortexed for 2 minutes. The emulsion obtained was centrifuged at 3600 rpm for 10 min. The tubes were placed in an ice bath, and the absorbance of the aqueous layer was obtained by spectrophotometric reading at 595 nm. The amount of blue dye extracted per gram of glandular tissue was then calculated. The result was expressed as mg Alcian/g tissue.

#### 2.8.2. Determination of Gastric Acid Secretion

This assay was performed according to the protocol described by Shay et al. [47], with slight modifications. The animals were divided into 4 groups and fasted for 16 hours with free access to 5% glycoside water solution. For pyloric ligation, the animals were anesthetized (xylazine 6 mg/kg associated with ketamine, 60 mg/kg, i.p.), the abdomen was opened for exposure of the stomach, and the pylorus was tied with a suture. Immediately after ligation, the animals received an intraduodenal solution of 0.9% NaCl (0.1 mL/100 g, negative control, *n* = 6), ranitidine (60 mg/kg, *n* = 7), or DELfp (200 mg/kg, *n* = 6). The abdominal wall was sutured, and four hours after pylorus ligation, the animals were euthanized. The noninjured control group (*n* = 5) was used in this experiment; animals of this group did not receive intraduodenal treatment but underwent surgical stress. One animal in this group died of anesthetic complications. Gastric secretion was collected and centrifuged at 1500 rpm for 30 min. Gastric content (g), pH, and total acidity (mEquiv. [H^+^]/mL/4 h) values were estimated.

#### 2.8.3. Involvement of Nitric Oxide (NO) and Sulfhydryl (-SH) Compounds in Gastroprotection

To investigate the influence of nitric oxide and endogenous sulfhydryl compounds on gastroprotection, male rats were distributed in 12 groups and remained in the fasted state for 16 hours. All groups were pretreated intraperitoneally, six groups received 0.9% NaCl solution (*n* = 6), three groups received N-nitro-L-arginine methyl ester (L-NAME, 70 mg/kg, i.p., *n* = 8), an inhibitor of the NO-synthase enzyme, and three groups received N-ethylmaleimide (NEM, 10 mg/kg, i.p., *n* = 6), a blocker of SH compounds responsible for the maintenance of the mucosal barrier integrity [48, 49]. After 30 min of pretreatment, 0.9% NaCl solution (control), carbenoxolone (100 mg/kg), or DELfp (200 mg/kg) was given orally. After 1 h, all animals received Merck absolute ethanol (≥99.9%, 1 mL, orally) for the induction of gastric ulcers. After 1 h of administration of the ulcerogenic agent, the animals were euthanized in a CO_2_ chamber. The stomachs were removed for the determination of the superficial area of the gastric lesion in the ImageJ software (Bethesda, MD, USA). The results were expressed in mm^2^.

### 2.9. Evaluation of the Healing Properties of DELfp

#### 2.9.1. Acetic Acid-Induced Gastric Ulcer

The induction of chronic ulcer was based on the study of Takagi et al. [50], with some modifications. The animals were divided into four groups and fasted for 16 hours. After fasting, the animals were anesthetized (xylazine, 6 mg/kg associated with ketamine, 60 mg/kg, i.p.) for surgical exposure of the stomach, and then, 0.05 mL of 30% acetic acid was injected in the subserous layer of the external wall of the stomach. One day after surgery, daily treatment was started and the animals were treated orally once a day for 14 consecutive days with 0.9% NaCl solution (negative control, *n* = 6), ranitidine (60 mg/kg, *n* = 6), or DELfp (200 mg/kg, *n* = 8). The noninjured control group (*n* = 6), in which the animals received no treatment but underwent surgical stress, with incision and suture of the abdominal wall, was used in this experiment. During treatment, the animals were observed for signs of toxicity such as piloerection, diarrhea, changes in locomotor activity, or mortality, and body weight was recorded. On day 15, the rats were euthanized, the stomachs were removed and photographed, and the lesions were measured in the ImageJ software (Bethesda, MD, USA). The results were expressed as the total area of ulcerative lesions (mm^2^).

#### 2.9.2. Histological Analysis

The stomachs with ulcers were sectioned and fixed in 10% buffered formalin. Once fixed, the samples were washed with water, immersed in 70% ethyl alcohol for 3-4 days, and embedded in paraffin. 5 *μ*m thick paraffin sections were fixed and stained with hematoxylin/eosin (H&E) and Masson's trichrome (TM) staining. The histological analysis of the gastric sections was made using a MICRO DIP automated microscopy system.

### 2.10. Evaluation of Anti-*Helicobacter pylori* Activity

The activity on bacterial growth was evaluated through minimum inhibitory concentration (MIC) by the broth microdilution technique and minimum bactericidal concentration (MBC) according to the standard established by the Clinical and Laboratory Standards Institute (CLSI) [51] with modifications [52]. The assays were performed with the *Helicobacter pylori* ATCC 43504 strain (sensitive to amoxicillin and resistant to metronidazole), grown initially on plates containing Columbia agar supplemented with 5% ram blood incubated for 72 hours at 37°C in an atmosphere containing 10% CO_2_. After that, the colonies were transferred to Brain Heart Infusion (BHI) broth (Merck Millipore cod. 110493, Darmstadt, Germany) supplemented with 10% (*v*/*v*) fetal bovine serum (Sigma-Aldrich cod. F2561; St. Louis, MO, USA) and incubated under the same conditions.

For the MIC assay, 100 *μ*L of different concentrations of the samples (32 to 1024 *μ*g/mL) was added to each well of a 96-well microplate and the same volume of bacterial suspension (dilution 1 : 20 of the 0.5 McFarland scale) in supplemented BHI. For bacterial growth control, the bacterial suspension and BHI medium were added; for the negative control, only samples and supplemented BHI medium were added. Growth inhibition was determined by the absorbance difference at 620 nm obtained before and after incubation for 72 hours at 37°C/10% CO_2_. CIM was graphically defined as the lowest concentration of samples that induced a sharp decline in absorbance (90% inhibition).

MBC was determined by the lower concentration of samples capable of inhibiting colony formation in Columbia Agar plates containing 5% sheep blood (incubated at 37°C/10% CO_2_ for 72 h) corresponding to the microplate wells of the MIC assay without apparent growth. Amoxicillin (Sigma-Aldrich cod. A8523; St. Louis, MO, USA) was used as the standard for anti-*H. pylori* control.

### 2.11. Statistical Analysis

Results were expressed as mean ± standard error of the mean (SEM). Differences between means were determined by analysis of variance (ANOVA) followed by Dunnett's multiple comparison test. The level of significance for rejection of the null hypothesis was 5% (*p* < 0.05). In the acute toxicity protocols and the influence of nitric oxide and sulfhydryl compounds on gastroprotection, the unpaired Student *t*-test was used to test for statistical differences between the two groups. Statistical analyses were performed in the GraphPad Prism version 7.0 (GraphPad Software, Inc., La Jolla, CA, USA).

## 3. Results and Discussion

### 3.1. Phytochemical Profile

#### 3.1.1. High-Performance Liquid Chromatography (HPLC-MS)

**Figure 1:**
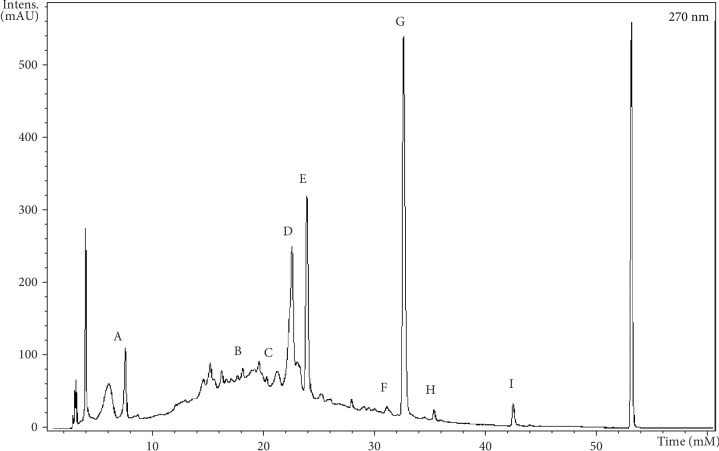
Chromatogram of the *L. ferrea* extract at 270 nm: (A) galloylquinic acid, (B) galloyl-HHDP-hex, (C) brevifolin carboxylic acid, (D) valoneic acid dilactone, (E) gallic acid derivative, (F) ellagic acid derivative (ellagic acid hex-), (G) ellagic acid, (H) ellagic acid derivative, and (I) dihydroisovaltrate.

Chromatographic analysis of the dry extract of *L. ferrea* pods suggested the presence of nine compounds: (A) galloylquinic acid (7.4 min), (B) galloyl-HHDP-hex (19.8 min), (C) brevifolin carboxylic acid (20.2 min), (D) valoneic acid dilactone (22.0 min), (E) gallic acid derivative (23.9 min), (F) ellagic acid derivative (ellagic acid hex-) (32.6 min), (G) ellagic acid (32.5 min), (H) ellagic acid derivative (35.3 min), and dihydroisovaltrate (42.3 min) (see Figure 1).

The compounds were identified by similarity between ultraviolet (UV) absorption spectra, mass/charge ratio, and fragmentation profile (MS^2^) with corresponding values in the literature (see Table 1).

According to the UV chromatogram at 270 nm, the main constituents of the DELfp are hydrolyzable tannins and phenolic compounds, since they have the same fragmentation profile described by Wyrepkowski et al. [53], Fischer et al. [54], Santos et al. [55], Sun et al. [56], Mullen et al. [57], and Abu-Reidah et al. [58].

Hydrolyzable tannins are readily hydrolyzed *in vivo* by the action of acids and/or enzymes releasing gallic acid and ellagic acid units [59, 60]. Gallic acid has anti-inflammatory and antioxidant activity, and ellagic acid has demonstrated antimutagenic and antioxidant activity [60].

Vasconcelos et al. [26] identified hydrolyzable tannins (gallic acid and ellagic acid) as the major compounds in the aqueous extract of the *C. ferrea* stem bark and related them to the reduction of oxidative stress in the hepatic tissue of diabetic rats.

**Figure 2:**
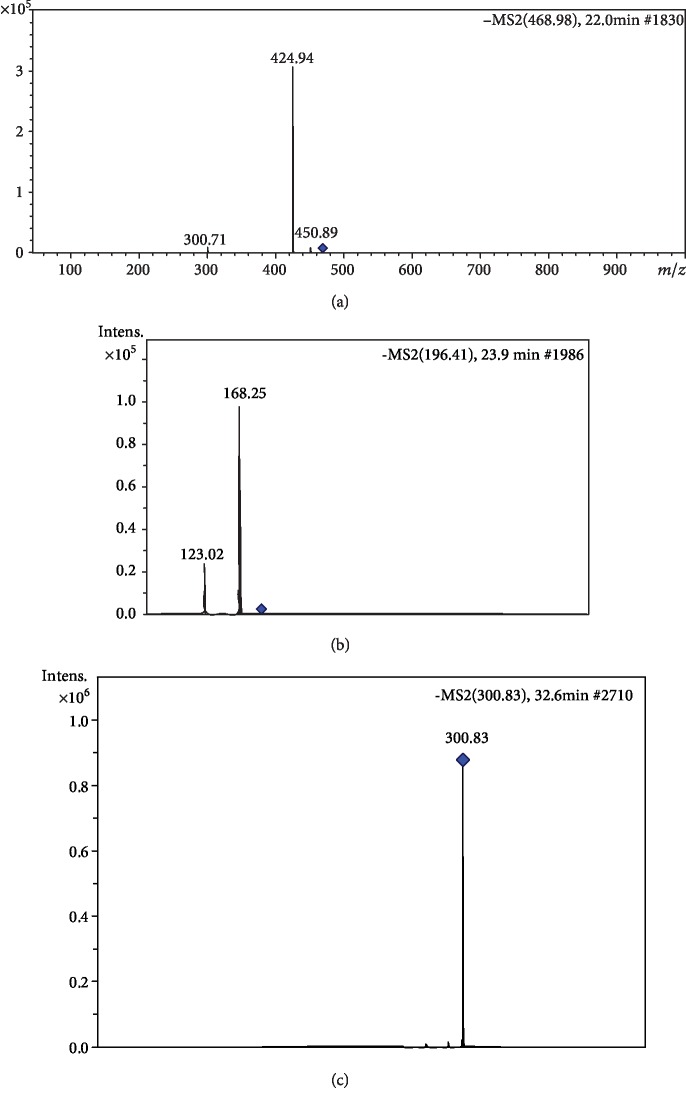
MS/MS spectra obtained from the chromatograms and the proposed identified compounds: (a) valoneic acid dilactone; (b) gallic acid derivative; (c) ellagic acid.

The presence of the valoneic acid dilactone (VAD) (D) was suggested in DELfp, with *m*/*z* 468.98 and fragmentation profile *m*/*z* 300.71, 424.94, and 450.89 (e.g., in Figure 2(a)). VAD presents in its structure etheric ellagic acid, by oxidative coupling with gallic acid [61].

The ion derived from deprotonation of the molecule at *m*/*z* 196.41 (E) was identified in Rt of 23.9 minutes, with fragmentation spectra *m*/*z* 168.25 and 123.02 (e.g., in Figure 2(b)). Wyrepkowski et al. [53] identified this compound as ethyl gallate, derived from gallic acid, in the chromatographic analysis of the *C. ferrea* stem bark.

Ellagic acid (G), observed at *m*/*z* 300.82, produced a *m*/*z* 256.63 fragment during fragmentation (e.g., in Figure 2(c)), which is in line with the results found by Wyrepkowski et al. [53] who also detected the *m*/*z* 301 ion as ellagic acid, with the fragmentation profile *m*/*z* 257 [M-H-CO_2_]^−^, *m*/*z* 229 [M-H-CO_2_-CO]^−^, and *m*/*z* 185 [M-H-2CO_2_-CO]^−^.

Brito et al. [62] found that gallic acid and ellagic acid are isolated gastric protectors, but when associated, these compounds act synergistically to protect the mucosa in preclinical models of gastric lesions.

The phytochemical analysis of DELfp allowed the quantification of total phenolic compounds and flavonoids and evaluation of the antioxidant and antiulcerogenic activity of the extract.

### 3.2. Quantification of Secondary Metabolite Classes in DELfp

Phenolic compounds exhibit antioxidant activity due to the ability to donate hydrogen or electrons and formation of stable intermediate radicals that prevent lipid oxidation [36]. The content of phenolic compounds was determined by an assay using the Folin-Ciocalteu reagent, where the intensity of the blue complex formed indicates the total content of phenolic compounds and is related to a greater number of hydrogenation groups present [63].

The quantification of total phenolic compounds in DELfp was 951.39 ± 0.01 mg EAG/g. Silva et al. [64] analyzed three plant species for antioxidant capacity and DNA protection and observed that *L. ferrea* fruits had the ability to eliminate the damage to DNA produced by hydroxyl radicals, and they associated this antioxidant activity with the high content of phenolic compounds of the species (460.00 ± 4.16 mg EAG/g).

Port's et al. [65] evaluated the phenolic compounds of *Chrysobalanus icaco* (51.30 mg EAG/g), *Lagerstroemia speciosa* (47.54 mg EAG/g), and *L. ferrea* (68.13 mg EAG/g) from the Amazon region. *L. ferrea* presented the highest content of polyphenols and the highest antioxidant capacity among the studied species.

Flavonoids represent a large group of low molecular weight phenolic substances with chemical structures and diverse characteristics [66]. They are responsible for protecting plants from ultraviolet (UV) radiation and phytopathogens [67]. They also present important biological activities, such as antioxidant activity, through elimination of free radicals [67], and gastroprotective activity, with antisecretory and cytoprotective activities [3]. The total flavonoid content in the DELfp was 35.35 mg EQ/g extract.

Flavonoids are known to inhibit the enzymatic activity of histidine decarboxylase [68] and thus reduce the formation of histamine in the gastric mucosa, a substance that directly stimulates parietal cells and pepsinogen responsible for the secretion of hydrochloric acid and pepsin, respectively [69].

Another substance involved in gastric damage is platelet-activating factor (PAF), an endogenous phospholipid that causes gastrointestinal ulceration [70] and predisposes the stomach to damage caused by ethanol directly applied to the gastric mucosa [71]. Intraperitoneal administration of quercetin, rutin, and kaempferol (25-100 mg/kg) inhibited the gastric content of PAF [69].

### 3.3. Antioxidant Activity *In Vitro*

Antioxidants protect the body against the oxidative damage involved in various diseases, which are believed to be caused or related to excess free radicals in the body, including cancer, hypertension, heart disease, and diabetes [72, 73]. Oxidative stress has a role in the etiology of gastrointestinal diseases. The increase in oxidizing agents and the overload of endogenous antioxidants favor the process of gastric ulceration [13].

Studies indicate that there is no single method capable of quantitatively and accurately evaluating antioxidant properties. As the various methodologies differ in their mechanisms of action, they are considered complementary in the study of the antioxidant potential of plants [74, 75].

In this study, different types of assays were used to evaluate the potential antioxidant of DELfp. The results on the inhibition of free radical at IC_50_ values are shown in Table 2. From the IC_50_ values described in the table, it is observed that both in the reduction of the DPPH radical and in the ABTS radical elimination capacity, the DELfp presented higher antioxidant power when compared to the Trolox standard. In addition, it also presented total antioxidant activity (TAA) and antioxidant activity to superoxide radicals (SRSA).

In the study of Port's et al. [65], *L. ferrea* leaves had a capacity to reduce the DPPH radical, presenting an IC_50_ value of 46.70 *μ*g/mL. Hassan et al. [76] found a remarkable scavenging activity of the DPPH radical by the ethanolic extract of *C. ferrea*, compared with ascorbic acid. The antioxidant capacity of the extract (IC_50_) was determined (12.45 ± 2.86 *μ*g/mL).

#### 3.3.1. Total Antioxidant Activity

The total antioxidant activity (%TAA) of DELfp at different concentrations (31.25-1000 *μ*g/mL) was 0.18 ± 0.04-76.10 ± 1.63%. Similarly, Silva et al. [64] found that the total antioxidant activity (%TAA) of the hydroalcoholic extract of fruits of *L. ferrea* was 38.06 ± 2.04% in relation to the activity of the standard, ascorbic acid.

#### 3.3.2. Superoxide Radical (O_2_^−^) Scavenging Activity

The extract of *L. ferrea* was able to scavenge the superoxide radical formed, presenting IC_50_ = 119.64 *μ*g/mL. This activity is important to prevent the damages generated by oxidative stress. Silva et al. [64] showed a significant and linear correlation between the phenolic content of *L. ferrea* fruits and the assays P-Mo and O_2_^−^ scavenging activity, with IC_50_ = 16.66 ± 2.91 *μ*g/mL by the method of Shukla et al. [77], thus indicating that the phenolic compounds are the main contributors to the antioxidant activity of the species.

The data obtained indicate that the DELfp presents medicinal potential and can be used in the prevention of several diseases associated with oxidative stress, through its antioxidant activity.

### 3.4. Acute Toxicity

Acute toxicity (LD_50_) is a test that assesses the toxic potential of a substance. Although this test can be used for plant extracts, there are peculiarities (e.g., place of collection of plant material, temperature, altitude, and soil) that should be taken into consideration, as they may influence the result. Therefore, we repeat the test, even knowing previous studies.

Administration of DELfp (2000 mg/kg, orally) did not cause death and behavioral changes during the 14 days of observation in female Wistar rats. Thus, LD_50_ was greater than 2000 mg/kg. The extract did not cause changes in the food consumption and body weight gain of the animals but led to higher water consumption (17.8%) compared to the control group (see Table 3).

Freitas et al. [78] evaluated in mice the acute toxicity of the crude extract and purified fraction of *L. ferrea* pods at doses of 300 and 2000 mg/kg and found no deaths or alterations in the evaluated parameters.

In our study, we observed increased water consumption in the DELfp group (2000 mg/kg). However, toxicological studies conducted in our laboratory (not yet published) showed that oral treatment for 30 days with DELfp (1000 mg/kg) in female rats did not change water consumption. In addition, it did not alter renal function indicators such as blood urea nitrogen, albumin, creatinine, and uric acid. Despite this information, it cannot be discarded that the presence of many secondary metabolites present in the extract (2000 mg/kg) can interfere with physiological mechanisms at a metabolic level or in the central nervous system inducing polydipsia. In this sense, further studies are needed to clarify the mechanism involved in increased water consumption.

Kobayashi et al. [79] evaluated in rats the acute toxicity of the ethanolic extract of *L. ferrea* pods (5000 mg/kg), and the results showed no death or signs of toxicity in the animals.

The gastroprotective activity of DELfp was evaluated by means of experimental models of absolute ethanol-, acidified ethanol-, and indomethacin- (NSAID) induced ulcers. These models are well described in the literature [15], and the lesions occur predominantly in the glandular portion of the stomach [80]. Reactive oxygen species contribute to the pathogenesis of these lesions, especially when they are caused by ethanol [81, 82].

### 3.5. Ethanol-Induced Ulcer

**Figure 3:**
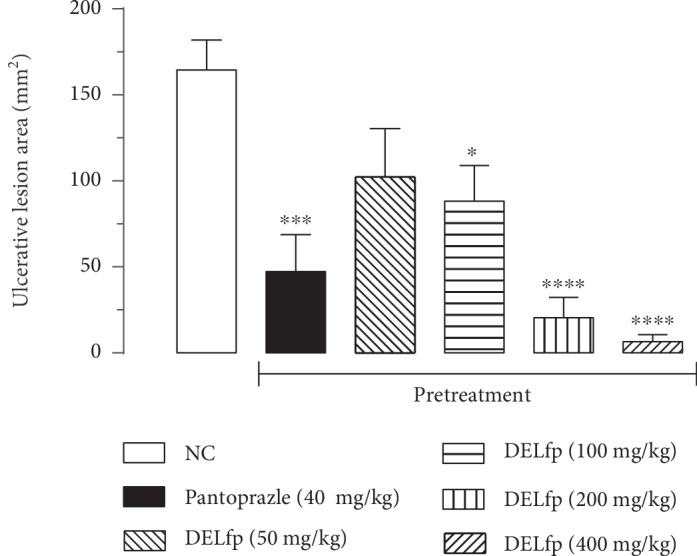
Gastroprotective effect of the dry extract of *L. ferrea* pods (DELfp) on absolute ethanol-induced gastric lesions in Wistar rats. The animals received, orally, 0.9% NaCl solution (negative control, NC), pantoprazole (40 mg/kg), or DELfp (50, 100, 200, and 400 mg/kg). Results are expressed as mean ± SEM (5-7 animals/group). Statistically different when compared to the negative control group, ANOVA followed by Dunnett's multiple comparison test; ^∗^*p* < 0.05, ^∗∗∗^*p* < 0.001, and ^∗∗∗∗^*p* < 0.0001.

**Figure 4:**
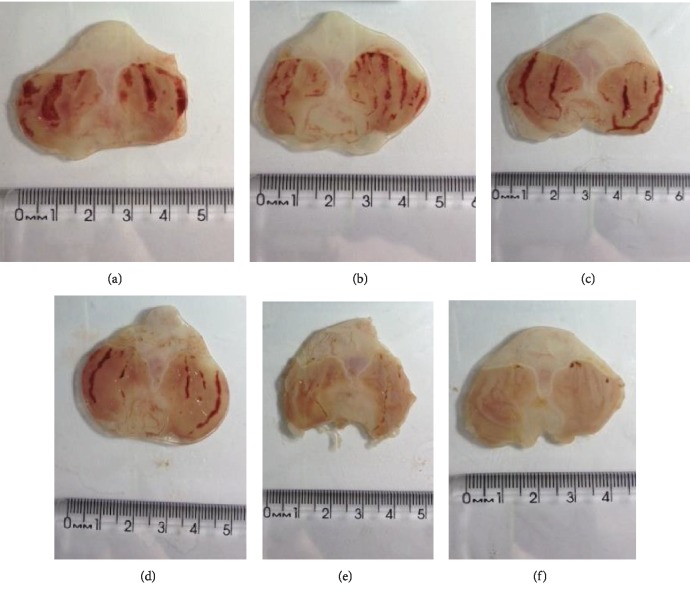
Typical photographs of the gastroprotective effect of the dry extract of *L. ferrea* pods (DELfp) on absolute ethanol-induced gastric lesions in Wistar rats. Top panel: (a) negative control, (b) pantoprazole (40 mg/kg), and (c) DELfp (50 mg/kg). Bottom panel: DELfp (100, 200, and 400 mg/kg, respectively) (d, e, f).

Administration of DELfp inhibited significantly the formation of absolute ethanol-induced lesions (see Figures 3 and 4) at 46.36, 87.56, and 95.99% in animals pretreated with the extract at the doses of 100, 200, and 400 mg/kg, respectively, when compared to the negative control group (NC, 164.50 ± 17.38 mm^2^). The ED_50_ value was 113.7 mg/kg. Rats treated with pantoprazole presented a gastroprotection of 71.20% of the injured area.

Oral administration of ethanol causes hemorrhagic lesions in the gastric mucosa, extensive submucosal edema, infiltration of inflammatory cells, and loss of epithelial cells [83]. The damage to the gastric mucosa caused by ethanol impairs microcirculation, damaging the endothelium of the capillary vessels and causing microvascular stasis with reduction of tissue oxygen [84, 85]. This ulcerogenic agent still inhibits the production of prostaglandins, decreases mucus production [8], and induces free radical formation, increasing lipid peroxidation in the mucosa [86].

Reactive oxygen species have a role in the pathogenesis of gastric ulcer, also when these lesions are caused by ethanol. Ethanol leads to increased superoxide anions, hydroxyl radicals, and lipid peroxidation in the gastric mucosa, inducing intracellular oxidative stress [81, 82, 87].

Pretreatment with DELfp (100, 200, and 400 mg/kg, orally) and pantoprazole (40 mg/kg) decreased lipid peroxidation levels by 44.19, 36.05, 44.19, and 43.03%, respectively, when compared to the negative control group (NC, 0.86 ± 0.12) (see Table 4). The chromatographic analysis of DELfp suggests the presence of compounds with antioxidant activity, which is promising in the treatment of gastric ulcers.

Malondialdehyde (MDA) is a marker of oxidative damage in physiological systems [88], resulting from lipid oxidation that occurs through a free-radical chain mechanism [89]. Caldas et al. [90] and Brito et al. [62] showed that compared to the levels observed in noninjured animals, animals subjected to ethanol-induced damage exhibited an increase in malondialdehyde (MDA) levels, as well as a reduction in the levels of nonprotein sulfhydryl (-SH) compounds.

GSH concentrations are physiologically elevated in gastric tissue, perhaps conferring some additional protection from the effects of gastric acid [91]. Oral administration of DELfp at the doses of 100 and 200 mg/kg significantly increased the endogenous antioxidant reserve (reduced glutathione, GSH) by 754.90 ± 72.48 (60.62%) and 766.60 ± 69.39 (63.11%) nmol of GSH/mg protein, respectively, when compared to the control group (470.00 ± 23.75) (see Table 4).

Rozza et al. [92] found that the pretreatment with the hydroalcoholic extract of the leaves of *Bauhinia holophylla* increased GSH levels by 65.5% in the ethanol-induced gastric ulcer model, since the etiological factors of gastric ulcers are closely associated with oxidative stress and the depletion of glutathione levels in the gastric mucosa [93]. In our study, the administration of the extract, prior to ethanol, also increased the concentration of sulfhydryl compounds and suggests an antioxidant activity of *L. ferrea* that prevents the oxidative effects of ethanol and contributes to its gastroprotective activity.

#### 3.5.1. Histopathological Analysis

**Figure 5:**
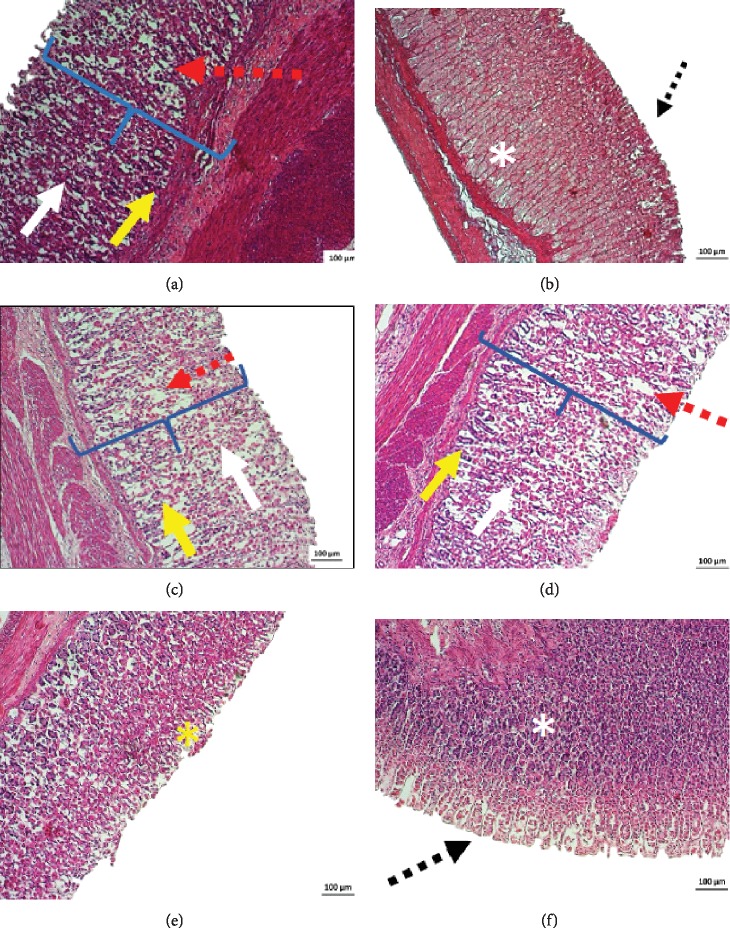
Histopathology of the gastric mucosa of rats from the experimental groups pretreated with 0.9% NaCl (negative control, a), pantoprazole (40 mg/kg, b), and dry extract of *L. ferrea* pods (DELfp 50, 100, 200, and 400 mg/kg; c, d, e, and f, respectively) in ethanol-induced ulcer. White arrows indicate disorganization of the simple columnar epithelium in the gastric cavities. Yellow arrows indicate congestion of blood capillaries. “{” indicates necrosis of the gastric mucosa. White asterisks (∗) indicate well-preserved gastric glands. The yellow asterisk (∗) indicates the unpreserved gastric mucosa with exfoliation of the simple columnar epithelium. Dotted black arrows indicate the preserved gastric mucosa, and dotted red arrows indicate edema.

In the histopathological analysis of the stomachs of the negative control group, it was possible to observe a disorganization of the simple columnar epithelium, capillary blood congestion, edema, and necrosis of the gastric mucosa (e.g., in Figure 5(a)). The pantoprazole group had a preserved gastric mucosa and glands (e.g., in Figure 5(b)).

In the DELfp group (50 and 100 mg/kg), it was possible to verify a disorganization of the simple columnar epithelium, capillary blood congestion, edema, and necrosis of the gastric mucosa (e.g., in Figures 5(c) and 5(d)). The DELfp group (200 mg/kg) presented an unpreserved gastric mucosa, exfoliation of the simple columnar epithelium, and edema. Congestion of blood capillaries and necrosis were not observed (e.g., in Figure 5(e)). DELfp (400 mg/kg) maintained the gastric mucus and glands preserved (e.g., in Figure 5(f)).

### 3.6. Acidified Ethanol- (Ethanol/HCl) Induced Ulcer

**Figure 6:**
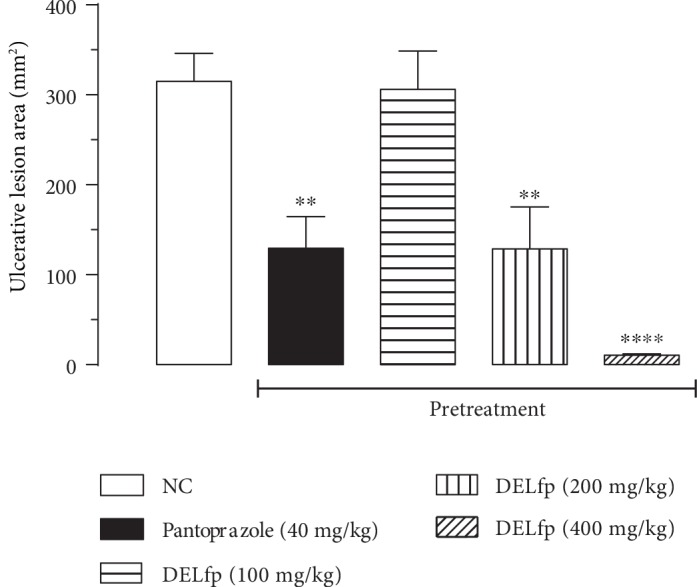
Gastroprotective effect of the dry extract of *L. ferrea* pods (DELfp) on ethanol/HCl-induced gastric lesions in Wistar rats. Animals received, orally, 0.9% NaCl (negative control, NC), pantoprazole (40 mg/kg), or DELfp (100, 200, and 400 mg/kg). Results are expressed as mean ± SEM (6-7 animals/group). Statistically different when compared to the negative control group, ANOVA followed by Dunnett's multiple comparison test; ^∗∗^*p* < 0.01 and ^∗∗∗∗^*p* < 0.0001.

**Figure 7:**
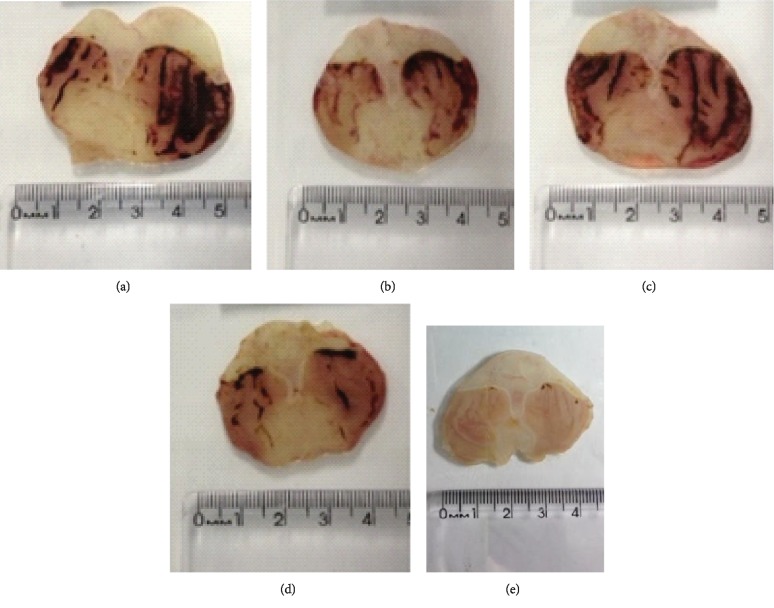
Typical photographs of the gastroprotective effect of the dry extract of *L. ferrea* pods (DELfp) on ethanol/HCl-induced gastric lesions in Wistar rats: (a) negative control, (b) pantoprazole (40 mg/kg), and (c, d, e) DELfp (100, 200, and 400 mg/kg, respectively).

The dry extract of *L. ferrea* pods (DELfp) promoted significant protection (200 and 400 mg/kg) against acidified ethanol- (ethanol/HCl) induced ulcer (see Figures 6 and 7). The results showed that animals pretreated at the doses mentioned showed inhibition of lesions of 59.12 and 96.61%, respectively, compared to the negative control group (NC, 314.80 ± 31.23 mm^2^). The ED_50_ value was 185.7 mg/kg. Likewise, animals treated with pantoprazole had a 58.93% reduction in the lesioned area.

Hydrochloric acid (HCl) causes stasis in blood flow and depresses the defense mechanisms of the gastric mucosa, enhancing the action of ethanol [94]. On the other hand, ethanol is considered one of the most intense agents in the induction of gastric lesions because it promotes serious mucosal disorders due to its direct action [95, 96]. Oral administration of DELfp (200 and 400 mg/kg) reduced ethanol/HCl-induced gastric lesions, indicating that *L. ferrea* has gastroprotective action.

### 3.7. Indomethacin-Induced Gastric Ulcer

**Figure 8:**
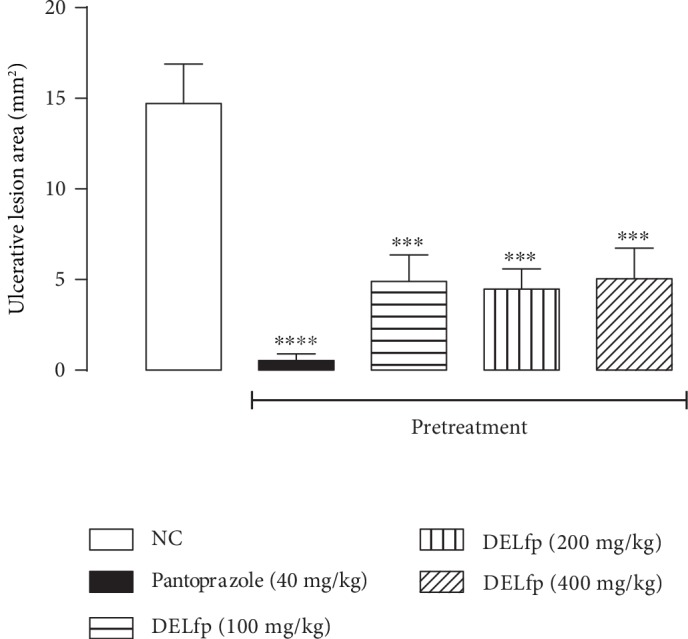
Gastroprotective effect of the dry extract of *L. ferrea* pods (DELfp) on indomethacin-induced gastric lesions (30 mg/kg, s.c.) in Wistar rats. Animals received, orally, 0.9% NaCl (negative control, NC), pantoprazole (40 mg/kg), or DELfp (100, 200, and 400 mg/kg). Results are expressed as mean ± SEM (6-7 animals/group). Statistically different when compared to the negative control group, ANOVA followed by Dunnett's multiple comparison test; ^∗∗∗^*p* < 0.001 and ^∗∗∗∗^*p* < 0.0001.

**Figure 9:**
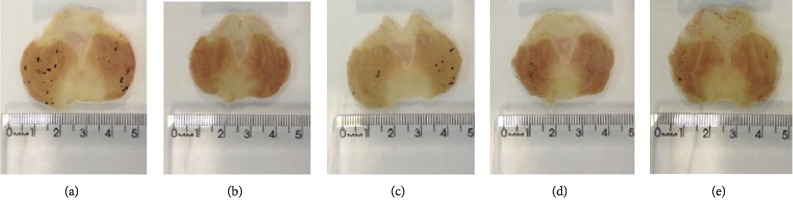
Typical photographs of the gastroprotective effect of the dry extract of *L. ferrea* pods (DELfp) on indomethacin-induced gastric lesions in Wistar rats: (a) negative control, (b) pantoprazole (40 mg/kg), and (c, d, e) DELfp (100, 200, and 400 mg/kg, respectively).

Gastric lesions induced by indomethacin (30 mg/kg), a nonsteroidal anti-inflammatory drug (NSAID), were significantly inhibited by DELfp (100, 200, and 400 mg/kg) at 66.72, 69.64, and 65.77%, respectively, compared to the gastric lesions (14.72 ± 2.18 mm^2^) of animals of the negative control group. In animals receiving pantoprazole, the lesioned area was inhibited by 96.40% (see Figures 8 and 9).

NSAID-induced gastric damages depend in part on their ability to reduce prostaglandin production by inhibiting cyclooxygenase (COX) and partly by COX-independent mechanisms [97]. The combined effects of these two mechanisms lead to marked oxidative tissue damage, which significantly contributes to gastric mucosal lesions [98].

In the stomach, prostaglandins play an important cytoprotective role because they stimulate the secretion of bicarbonate and mucus and maintain blood flow to the mucosa [97]. Hydrolyzable tannins and derivatives such as gallic acid, present in DELfp, have anti-inflammatory activity, preferentially inhibiting COX-2 [99].

Oral pretreatment with DELfp (100, 200, and 400 mg/kg) reduced the gastric lesions caused by indomethacin, suggesting possible involvement of prostaglandins and/or mucus in the antiulcerogenic activity of the extract.

However, other mechanisms may contribute to the gastroprotective effect of DELfp. Pereira et al. [100] reported significant anti-inflammatory activity of polysaccharide fractions of *L. ferrea* pods in carrageenan-induced edema via modulation of histamine, serotonin, bradykinin, and PGE_2_.

### 3.8. Protective Factors of the Gastric Mucosa

Our results showed that the ED_50_ values were 113 and 185.7 mg/kg, respectively, in the gastroprotection experiments (alcohol-induced and alcohol/HCl-induced ulcers). However, in the histological analysis from the ethanol-induced ulcer test, we found out that the histological parameters of the dose of 100 mg/kg (disorganization of the simple columnar epithelium in the gastric cavities, congestion of blood capillaries, necrosis of the gastric mucosa, and edema) were similar to those of the negative control, which led us to select the intermediate dose of 200 mg/kg to evaluate the possible mechanism of action. In fact, Adinortey et al. [101] have reported that the ethanol-induced ulcer model is not appropriate for the assessment of the usefulness of antisecretory drugs due to the absence of gastric secretion in this model.

#### 3.8.1. Effect of DELfp on Gastric Mucus Production

**Figure 10:**
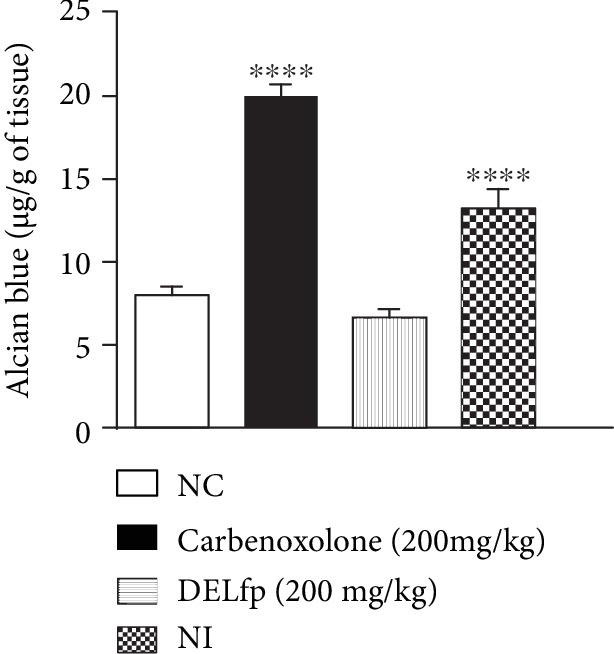
Effect of the dry extract of *L. ferrea* pods (DELfp) on mucus quantification. The negative control group (NC) received 0.9% NaCl; the positive control group received carbenoxolone (200 mg/kg), and the DELfp group received DELfp (200 mg/kg). The noninjured group (NI) received no treatment. Results are expressed as mean ± SEM (6-8 animals/group). Statistically different when compared to the negative control group, ANOVA followed by Dunnett's multiple comparison test; ^∗∗∗∗^*p* < 0.0001.

Pyloric ligation in rats of the negative control group resulted in a significant decrease in gastric mucus levels (NC, 7.88 ± 0.55 *μ*g Alcian blue/g tissue) compared to the noninjured group (NI, 13.35 ± 0.95 *μ*g of Alcian blue/g tissue). Rats treated with DELfp (200 mg/kg) had no increase in mucus levels (6.52 ± 0.38 g Alcian blue/g tissue) compared to the negative control group. However, carbenoxolone (200 mg/kg) promoted a significant increase in mucus levels of 148.92% over the negative control group (see Figure 10).

Carbenoxolone maintains the prostaglandin content of the gastric mucosa at high levels due to its inhibitory action on the catabolic enzymes 15-hydroxy-prostaglandin dehydrogenase and D13-prostaglandin-reductase, in addition to increasing AMP levels by inhibition of mucosal phosphodiesterases. Carbenoxolone is a cytoprotective agent because high levels of prostaglandins promote mucosal defense against ulceration [102]. In this study, carbenoxolone increased the concentration of Alcian blue; however, this effect was not seen with DELfp. Thus, the gastroprotective effect of the extract seems to be independent of mucus participation.

Beserra et al. [103] found that ellagic acid (EA) alone did not have a significant effect on mucus production in stomachs of animals submitted to ulceration. This data suggests that mucus production is not involved in the gastroprotective activity of EA. The gastroprotective properties of EA were partially attributed to the inhibitory action on the proton pump (H^+^, K^+^-ATPase) [104] and antioxidant properties in vivo [105]. The chromatographic analysis of DELfp showed the presence of ellagic acid and its derivatives in its composition. This may explain the noninvolvement of DELfp in the production of mucus.

#### 3.8.2. Effect of DELfp on Gastric Acid Secretion

After 4 h of pyloric ligation, it was observed that intraduodenal administration of DELfp (200 mg/kg) and ranitidine (60 mg/kg) reduced gastric secretion by 52.71 and 45.95%, respectively, and total acidity by 29.85 and 54.96%, respectively (see Table 5).

The pyloric ligation method promotes vagovagal reflexes, increasing gastric secretion through the stimulation of mechanoreceptors present in the antral mucosa, during the pylorus ligation [106]. The extract of *L. ferrea* decreased the acid secretion and the concentration of H^+^ in the gastric content. Endogenous histamine formation and release by mast cells are related to the pathogenesis of gastric ulcers produced by pylorus ligation, suggesting that antihistaminic (H_2_) drugs may be useful in the prevention of lesions [107].

The results suggested that one of the mechanisms of DELfp relates to antisecretory activity and confirmed the systemic effect of the extract in the treatment of gastric ulcers, in view of the effectiveness of the treatment when the extract was administered intraduodenally.

#### 3.8.3. Involvement of Nitric Oxide (NO) and Sulfhydryl (-SH) Compounds in Gastroprotection

N-Nitro-L-arginine methyl ester (L-NAME) and N-ethylmaleimide (NEM) exacerbated the gastric lesions induced by ethanol in 44.02 and 92.73%, respectively, in relation to the effects in the groups pretreated with NaCl solution. DELfp (200 mg/kg) presented a gastroprotective effect in the presence of L-NAME (70 mg/kg, i.p.), reducing the lesions by 95.58% compared to the blocked control group (196.30 ± 28.11 mm^2^). This result suggested that the gastroprotective effect of DELfp does not depend on the presence of nitric oxide (NO) (see Table 6). However, in the presence of NEM (10 mg/kg, i.p.), the gastroprotective effect of DELfp was not evident (399.80 ± 50.35 mm^2^) when compared to the negative control group (NC, 389.90 ± 38.15 mm^2^). Depletion of sulfhydryl compounds by pretreatment with NEM was able to eliminate the gastroprotective effect of DELfp against ulcerogenic agents. This indicates that the gastroprotective effect of DELfp depends entirely on the presence of sulfhydryl (-SH) compounds (see Table 6).

In this study, the data demonstrated that gastric exposure to ethanol associated with the depletion of sulfhydryl compounds promoted by NEM (SH inhibitor) and the inhibition of nitric oxide synthase (NOS) by L-NAME reduced gastroprotection.

Nitric oxide is considered one of the most important agents of defense of the gastric mucosa due to the increase in tissue blood flow [108]. DELfp presented compounds involved in gastroprotection, acting independently of the presence of nitric oxide but depending on the presence of sulfhydryl groups that are responsible for increasing mucus production, maintaining gastric integrity, and reducing free radical production [109].

### 3.9. Acetic Acid-Induced Gastric Ulcer

**Figure 11:**
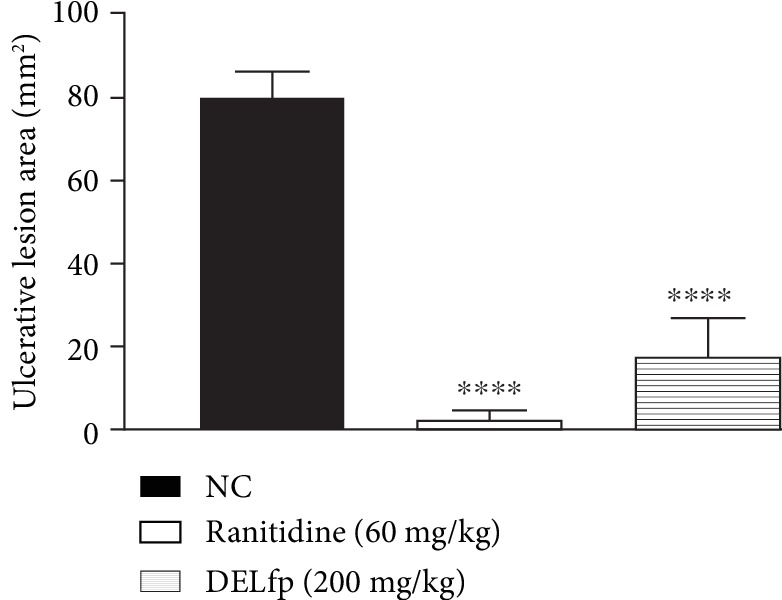
Effect of the dry extract of *L. ferrea* pods (DELfp) on the healing of the gastric mucosa in Wistar rats. Animals received, orally, 0.9% NaCl (negative control, NC), ranitidine (60 mg/kg), or DELfp (200 mg/kg). Results are expressed as mean ± SEM (*n* = 6-8/group). Statistically different when compared to the negative control group, ANOVA followed by Dunnett's multiple comparison test; ^∗∗∗∗^*p* < 0.0001.

**Figure 12:**
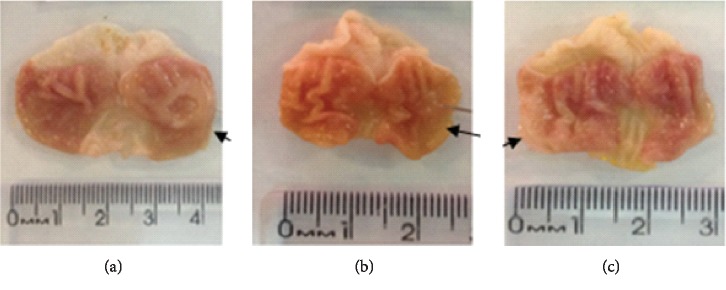
Typical photographs of the healing effect of the dry extract of *L. ferrea* pods (DELfp) on acetic acid-induced gastric ulcer in Wistar rats: (a) negative control group, (b) ranitidine (60 mg/kg), and (c) DELfp (200 mg/kg).

Our results showed that treatment with DELfp (200 mg/kg) and ranitidine (60 mg/kg) for 14 consecutive days reduced the area of chronic ulcers by 77.44 and 96.59% compared to the group treated with 0.9% NaCl solution (control), in which the injured area corresponded to 79.92 ± 6.53 mm^2^ (see Figures 11 and 12). It was possible to observe that DELfp showed healing activity of the gastric ulcer induced by acetic acid. The false-operated group, consisting of animals in which the ulcer was not induced, presented no lesion, as expected.

Administration of DELfp (200 mg/kg) and ranitidine (60 mg/kg) for 14 consecutive days did not cause visible signs of toxicity (diarrhea, piloerection, or changes in locomotor activity). A reduction of 6.89 and 7.17% in feed intake was observed in the animals treated with DELfp and ranitidine during 14 days (see Table 7).

Mangan [110] reported the astringent character of tannins when added to food or beverages. However, studies indicate that the major effect of tannins is not their inhibition in food consumption, but rather their decreased intestinal conversion and absorption of nutrients [111].

Oral administration of antihistamines (H_2_) such as ranitidine delays gastric emptying and causes dyspeptic symptoms such as early satiety, nausea, and abdominal swelling [112]. This fact may explain the reduction of feed consumption of the animals treated with ranitidine (60 mg/kg) for 14 consecutive days in this study.

Animals treated with DELfp also had 11.16% reduced water consumption compared to the control group (see Table 8). Body weight of the animals treated with DELfp or ranitidine was similar to that of the control group.

Administration of DELfp (200 mg/kg) for 14 consecutive days resulted in 5.08% reduction (16.44 ± 0.24) in mean corpuscular hemoglobin (MCH) compared to the control group (17.32 ± 0.18). In fact, a study conducted in our laboratory with prolonged administration (30 days) of DELfp (100 and 500 mg/kg) showed a reduction of 7.68% and 7.30% in HCM compared to the control group (unpublished data). Hematological changes may suggest chemical stress resulting from metabolic reactions caused by toxic substances [113]. The other hematological parameters and the biochemical parameters evaluated were not affected by DELfp (see Table 8).

#### 3.9.1. Histological Analysis

**Figure 13:**
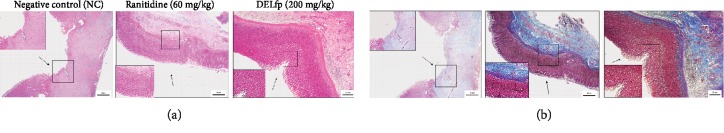
Photomicrographs of the gastric mucosa stained with hematoxylin/eosin (a) and Masson's trichrome (b) of rats submitted to induction of chronic ulcer by 30% acetic acid. The animals were treated orally with 0.9% NaCl solution (negative control, NC), ranitidine (60 mg/kg), or dry extract of *L. ferrea* pods (DELfp, 200 mg/kg) for 14 days. The filled arrow indicates the absence of ulcer and/or preserved columnar simple epithelium or reepithelialisation, and the dashed arrow indicates the presence of ulcer, lymphoplasmacytic infiltrate, and destruction of the mucosa.

In the histological analysis of the stomachs, sections fixed and stained with H&E and Masson's trichrome (TM) revealed well-defined ulcers with destruction of the mucosa and submucosa layer caused by acetic acid in animals of the control group. Stomachs of rats orally treated with DELfp (200 mg/kg) and ranitidine (60 mg/kg) presented similar tissue regeneration to those in the negative control group (see Figure 13).

### 3.10. *Helicobacter pylori*

The MIC of the extract was evaluated and, by definition, represents the concentration where at least 90% inhibition of bacterial growth was obtained. DELfp showed anti-*H. pylori* activity with MIC of 512 *μ*g/mL. Amoxicillin had an MIC of 0.3125 *μ*g/mL. In turn, MBC corresponded to the concentration where bacterial colonies were not observed. In this assay, the DELfp presented MBC of 512 *μ*g/mL.

The ethanolic extract of *Spondias mombin*, rich in gallic acid and ellagic acid, showed anti-*H. pylori* activity with MIC of 256 *μ*g/mL [62]. Other studies have demonstrated antibacterial activity of *L. ferrea* stem bark extracts against other bacterial strains such as *Staphylococcus aureus* [29, 100]. Baydar et al. [114] and Tomás-Menor et al. [115] showed that the antibacterial activity of plant species is proportional to the amount of phenolic compounds in the plant.

*Helicobacter pylori* infection induces inflammation, causing damage that is partly attributable to ROS production. The infection overwhelms the ability of mucosal cells and local glutathione to prevent ROS-mediated damage. Therapeutic regulation of glutathione availability prevents damage caused by *H. pylori* [91]. DELfp increased GSH levels in the gastric mucosa in ethanol-induced lesions, thus presenting potential to reduce *H. pylori* damage in the production of lesions.

## 4. Conclusions

DELfp showed gastroprotective, antiulcerogenic, and gastric healing activity mediated by antioxidant and antisecretory activity and the involvement of sulfhydryl compounds in addition to anti-*H. pylori* effect. These mechanisms may contribute to the healing of chronic ulcers, promoted by the dry extract of pods of *L. ferrea.* These data validate its use in folk medicine.

## Figures and Tables

**Table 1:** Data of the compounds detected by HPLC-MS from the dry extract of *L. ferrea* pods (negative mode).

Peak (compound)	Rt (min)	*λ*max (nm)	LC-MS	MS/MSn ions	Identification	Reference
			[M-H]^−^*m*/*z*			
	3.8	270	649.13	398.95/478.93/561.02/605.03	NI	—
A	7.4	214, 270	343.04	168.28/190.38	Galloylquinic acid	
						Wyrepkowski et al. [53]
B	19.7	272	633.08	185.30/300.74/336	Galloyl-HHDP-hex	Fischer et al. [54]
C	20.2	275	290.85	246.58	Brevifolin carboxylic acid	Fischer et al. [54]; Santos et al. [55]
D	22.3	256, 364	468.98	300.79/424.95/284.65	Dilactone of valoneic acid	Wyrepkowski et al. [53]
E	23.9	214, 270	196.41	168.25/290.80	Derivatives from gallic acid	Sun et al. [56]; Fischer et al. [54]; Wyrepkowski et al. [53]
F	31.2	—	463.09	300.83	Derivative of ellagic acid (ellagic acid hex)	Fischer et al. [54]
G	32.5	253, 367	300.82	256.63	Ellagic acid	Mullen et al. [57]; Wyrepkowski et al. [53]
H	35.3	—	497.05	300.78/450.92	Derivative of ellagic acid	Wyrepkowski et al. [53]
I	42.3	270	423	270	Dihydroisovaltrate	Abu-Reidah et al. [58]
	53.2	282	445.12	297.88/369.99/265.74	NI	—

**Table 2:** In vitro antioxidant activity IC_50_ (*μ*g/mL) of the dry extract of *L. ferrea* pods (DELfp) and antioxidant standards.

Sample	DPPH^+^ IC_50_ (*μ*g/mL)	ABTS^+^ IC_50_ (*μ*g/mL)	TAA IC_50_ (*μ*g/mL)	SRSA IC_50_ (*μ*g/mL)
DELfp	28.96	145.10	633.60	119.64
Trolox	53.20	199.27	NT	NT
Ascorbic acid	NT	NT	500.00	NT

DPPH: 2,2-diphenyl-1-picrylhydrazyl radical; ABTS: 2′,2-azino-bis(3-ethylbenzthiazoline-6-sulfonate) radical; TAA: total antioxidant activity; SRSA: superoxide radical scavenging assay; NT: not tested.

**Table 3:** Effect of the dry extract of *L. ferrea* pods (DELfp) on food consumption, water consumption, and body weight in female rats for 14 days.

Parameter	Control (0.9% NaCl)	DELfp (2000 mg/kg)
Food consumption (g)	16.75 ± 1.86	17.98 ± 0.71
Water consumption (mL)	31.96 ± 1.05	37.64 ± 1.19^∗^
Initial body weight (g)	216.70 ± 10.09	212.70 ± 18.70
Final body weight (g)	222.00 ± 4.61	222.70 ± 17.24

Values represent the mean ± SEM (*n* = 3/group). Statistically different when compared to the control group, Student's *t*-test for unpaired samples; ^∗^*p* < 0.02.

**Table 4:** Effect of oral pretreatment with the dry extract of *L. ferrea* pods (DELfp) on absolute ethanol-induced gastric lesions in Wistar rats.

Pretreatment	LPO (nmol of MDA/mg protein)	SH groups (nmol/mg protein)
Negative control (NC)	0.86 ± 0.12	470.00 ± 23.75
Pantoprazole (40 mg/kg)	0.49 ± 0.06^∗^	861.70±44.80^∗∗∗∗^
DELfp (100 mg/kg)	0.48±0.07^∗∗^	754.91±72.48^∗∗^
DELfp (200 mg/kg)	0.55 ± 0.04^∗^	766.60±69.39^∗∗^
DELfp (400 mg/kg)	0.48 ± 0.08^∗^	409.70 ± 23.04

LPO: lipid peroxidation; SH groups: nonprotein sulfhydryl groups. Results are expressed as mean ± SEM (*n* = 6-7/group). Statistically different when compared to the negative control group, ANOVA followed by Dunnett's multiple comparison test; ^∗^*p* < 0.05, ^∗∗^*p* < 0.01, and ^∗∗∗∗^*p* < 0.0001.

**Table 5:** Effect of the dry extract of *L. ferrea* pods (DELfp) on parameters of gastric secretion in Wistar rats submitted to pyloric ligation.

Treatment	pH	[H^+^] (mEq/mL/4 h)	Gastric content (g)
Noninjured	4.38 ± 0.51^∗^	10.78±1.04^∗∗^	0.21±0.02^∗∗∗∗^
Negative control	3.24 ± 0.10	18.63 ± 1.65	0.74 ± 0.08
Ranitidine (60 mg/kg)	3.83 ± 0.14	8.39±0.89^∗∗∗∗^	0.40±0.07^∗∗^
DELfp (200 mg/kg)	3.39 ± 0.09	13.07 ± 0.80^∗^	0.35±0.05^∗∗^

Values represent the mean ± SEM (5-7 animals/group). Statistically different when compared to the negative control group, ANOVA followed by Dunnett's multiple comparison test; ^∗^*p* < 0.05, ^∗∗^*p* < 0.01, and ^∗∗∗∗^*p* < 0.0001.

**Table 6:** Effect of oral administration of the dry extract of *L. ferrea* pods (DELfp) on ethanol-induced gastric lesions in Wistar rats pretreated with N-methyl-nitro-L-methyl arginine ester (L-NAME, 70 mg/kg) or N-ethylmaleimide (NEM, 10 mg/kg).

Pretreatment	Treatment (p.o.)	Dose (mg/kg)	Lesion area (mm^2^)	Inhibition (%)
0.9% NaCl (i.p.)	NC	—	136.30 ± 16.74	—
	Carbenoxolone	100	39.39 ± 8.64^∗^	71.11
	DELfp	200	15.49±6.69^∗∗^	88.64

L-NAME (i.p.)	NC	—	196.30 ± 28.11	—
	Carbenoxolone	100	116.30 ± 25.24	40.76
	DELfp	200	8.66±3.33^∗∗∗∗^	95.59

0.9% NaCl (i.p.)	NC	—	202.30 ± 32.37	—
	Carbenoxolone	100	29.56±5.88^∗∗∗^	85.39
	DELfp	200	8.04±4.84^∗∗∗^	96.03

NEM (i.p.)	NC	—	389.90 ± 38.15	—
	Carbenoxolone	100	32.26±10.17^∗∗∗∗^	91.73
	DELfp	200	399.80 ± 50.35	—

Results are expressed as mean ± SEM (*n* = 6-8 animals/group). NC: negative control group. Statistically different when compared to the negative control group, ANOVA followed by Dunnett's multiple comparison test; ^∗^*p* < 0.05, ^∗∗^*p* < 0.01, ^∗∗∗^*p* < 0.001, and ^∗∗∗∗^*p* < 0.0001.

**Table 7:** Effect of the dry extract of *L. ferrea* pods (DELfp) on feed and water intake and body weight of rats after 14 days of treatment in the acetic acid-induced ulcer model.

Parameter	Control (0.9% NaCl)	Ranitidine (60 mg/kg)	DELfp (200 mg/kg)
Food consumption (g)	287.60 ± 1.27	267.00±4.75^∗∗^	267.80±3.35^∗∗^
Water consumption (mL)	512.60 ± 10.07	480.90 ± 16.48	455.40±3.70^∗∗^
Initial body weight (g)	280.00 ± 4.53	275.70 ± 14.21	312.50 ± 9.69
Final body weight (g)	312.70 ± 3.78	302.00 ± 16.69	329.50 ± 10.03

Values represent the mean ± SEM (*n* = 6-8/group). Statistically different when compared to the control group, ANOVA followed by Dunnett's multiple comparison test; ^∗∗^*p* < 0.01.

**Table 8:** Effect of the dry extract of *L. ferrea* pods (DELfp) on hematological and biochemical parameters in rats after 14 days of treatment in the acetic acid-induced ulcer model.

Parameter	Control (0.9% NaCl)	Ranitidine (60 mg/kg)	DELfp (200 mg/kg)
Hematological parameter			
RBC (millions/mm^3^)	7.81 ± 0.14	7.28 ± 0.48	7.41 ± 0.29
Hb (g/dL)	12.75 ± 0.11	11.70 ± 0.68	12.09 ± 0.54
Ht (%)	43.13 ± 2.39	40.37 ± 2.73	40.64 ± 1.59
MCV (micra^3^)	54.50 ± 0.50	52.53 ± 2.28	54.88 ± 0.29
MCH (pg)	17.32 ± 0.18	17.12 ± 0.07	16.44 ± 0.24^∗^
MCHC (%)	34.50 ± 0.42	34.40 ± 0.37	32.48 ± 1.35
RDW (%)	13.58 ± 0.16	14.00 ± 0.29	13.40 ± 0.36
Platelets (mm^3^)	952.20 ± 39.24	1020.00 ± 45.51	924.60 ± 17.58
Biochemical parameter			
Creatinine (mg/dL)	0.78 ± 0.03	0.66 ± 0.04	0.81 ± 0.02
BUN (mg/dL)	42.67 ± 2.97	45.67 ± 1.47	43.13 ± 1.48
ALP (U/L)	230.50 ± 69.72	168.70 ± 25.20	144.60 ± 11.57
AST (U/L)	129.00 ± 14.06	168.00 ± 10.00	129.40 ± 5.78
ALT (U/L)	51.00 ± 5.93	51.00 ± 3.05	43.88 ± 2.83

RBC: red blood cell; Hb: hemoglobin; Ht: hematocrit; MCV: mean corpuscular volume; MCH: mean corpuscular hemoglobin; MCHC: mean corpuscular hemoglobin concentration; RDW: red cell distribution width; ALP: alkaline phosphatase; AST: aspartate aminotransferase; ALT: alanine aminotransferase; BUN: blood urea nitrogen. Values represent the mean ± SEM (*n* = 6-8/group). Statistically different when compared to the control group, ANOVA followed by Dunnett's multiple comparison test; ^∗^*p* < 0.05.

## Data Availability

We, the authors, support and endorse the FAIR Guiding Principles for scientific data management and stewardship—findability, accessibility, interoperability, and reusability. The experimental data used to support the findings of this study are included within the manuscript. However, additional information from this study may be obtained upon request to the corresponding author (almir.wanderley@ufpe.br or almirgw.wanderley@gmail.com). We ensured that Hindawi (*Oxidative Medicine and Cellular Longevity*) has the rights necessary for the proper administration of electronic rights and online dissemination of the manuscript entitled “Antioxidant and Antiulcerogenic Activity of the Dry Extract of Pods of *Libidibia ferrea* Mart. ex Tul. (Fabaceae).”
